# Health burden and economic impact of measles-related hospitalizations in Italy in 2002–2003

**DOI:** 10.1186/1471-2458-7-169

**Published:** 2007-07-24

**Authors:** Antonietta Filia, Antonio Brenna, Augusto Panà, Gianluca Maggio Cavallaro, Marco Massari, Marta L Ciofi degli Atti

**Affiliations:** 1 Centro Nazionale di Epidemiologia, Sorveglianza e Promozione della Salute, Istituto Superiore di Sanità, Viale Regina Elena, 299, 00161 Rome, Italy; 2 Dipartimento di Sanità Pubblica, Cattedra di Economia Sanitaria, Università Tor Vergata, Rome, Italy; 3 Dipartimento di Sanità Pubblica, Cattedra di Igiene, Università Tor Vergata, Rome, Italy

## Abstract

**Background:**

A large measles outbreak occurred in Italy in 2002–2003. This study evaluates the health burden and economic impact of measles-related hospitalizations in Italy during the specified period.

**Methods:**

Hospital discharge abstract data for measles hospitalizations in Italy during 2002–2003 were analysed to obtain information regarding number and rates of measles hospitalizations by geographical area and age group, length of hospital stay, and complications. Hospitalization costs were estimated on the basis of Diagnosis-Related Groups.

**Results:**

A total of 5,154 hospitalizations were identified, 3,478 (67%) of which occurred in children <15 years of age. Most hospitalizations occurred in southern Italy (71 %) and children below 1 year of age presented the greatest hospitalization rates (46.2/100,000 and 19.0/100,000, respectively in 2002 and 2003). Pneumonia was diagnosed in 594 cases (11.5%) and encephalitis in 138 cases (2.7%). Total hospital charges were approximately € 8.8 million.

**Conclusion:**

The nationwide health burden associated with measles during the 2002–2003 outbreak was substantial and a high cost was incurred by the Italian National Health Service for the thousands of measles-related hospitalizations which occurred. By assuming that hospital costs represent 40–50% of the direct costs of measles cases, direct costs of measles for the two years combined were estimated to be between €17.6 – 22.0 million, which equates to the vaccination of 1.5–1.9 million children (3–4 birth cohorts) with one dose of MMR. The high cost of measles and the severity of its complications fully justify the commitment required to reach measles elimination.

## Background

In Italy, measles vaccine was first introduced in 1979 and combined measles-mumps-rubella (MMR) vaccines became available in the early 1990s. Initially, routine administration of one dose of measles vaccine to all children aged ≥ 15 months was recommended, while the current two dose schedule (one dose at age 12–15 months and another at 5–6 years or 11–12 years) was introduced in 1999 [1].

Despite the existence of national recommendations, however, each of the country's 21 regions was responsible for implementation of strategies regarding non-compulsory vaccinations, including measles. This contributed to non uniform vaccine coverage rates observed across Italy, with lower rates observed mainly in southern regions [2]. National coverage for one dose of MMR vaccine in children aged 12–24 months also remained inadequate and was estimated to be 56% in 1998 [3] and 74% in 2001 [4].

Due to suboptimal coverage rates, a large proportion of individuals remained susceptible to measles and in 2002, a large outbreak occurred in Campania, a region of southern Italy [5]. The outbreak then spread to other regions, prevalently in southern Italy, with an estimated national incidence, in children below 15 years of age, of 738/100,000 in 2002 and 544/100,000 in 2003, corresponding to over 100,000 estimated cases in this age group [6].

It is well known that measles infection can cause serious complications and between 1.4% and 19.0% of measles cases that occur in industrialized countries require hospitalization [7-11]. Many measles-related hospitalizations were therefore expected to have occurred across Italy in 2002 and 2003.

In addition to their disease burden, hospitalized measles cases impose an economical burden to the healthcare system and to society as a whole. The majority of economic costs associated with measles have, in fact, been attributed to the cost of hospitalizing patients [10]. In order to obtain information on the health burden and economic impact of a measles outbreak in Italy, therefore, we evaluated national discharge data regarding measles-related hospitalizations that occurred in the years 2002–2003.

## Methods

### Sources of data

Hospital discharge abstract data for measles-related hospitalizations that occurred in Italy between January 1st 2002 and December 31st 2003, was obtained from the national discharge abstract data bank, held by the Ministry of Health. This database contains administrative and health data regarding hospital admissions, that all public and privately-owned hospitals in Italy are legally required to report [12].

For each admission, a main discharge diagnosis is reported; this represents the clinical condition which took up the greatest amount of resources and therefore involved the greatest cost for the hospital. Up to five additional secondary diagnoses may be listed.

Diagnoses are coded by using International Classification of Diseases-Clinical Modification, 9^th ^edition (ICD9-CM) nomenclature. Measles-related codes include the following: postmeasles encephalitis (055.0), postmeasles pneumonia (055.1), postmeasles otitis media (055.2), measles keratoconjunctivitis (055.71), measles with other specified complication (055.79), measles with unspecified complication (055.8) and measles without mention of complication (code 055.9).

In this study, we included all admissions with at least one measles-related main or secondary discharge diagnosis. For each of these hospitalizations, we obtained the following data: hospital code, age, gender, municipality, province and region codes, citizenship, admission and discharge dates, type of admission (i.e. emergency or ordinary), payer of hospital expenses (i.e. National Health Service or other), discharge status (i.e. death, ordinary or voluntary discharge, transferred to other acute care hospital), main discharge diagnosis, secondary diagnoses (if present), Diagnosis Related Group (DRG).

Data provided by the Ministry of Health did not contain any patient identifiers and was therefore completely anonymous. Ethical approval was therefore not required for this study [13].

Population data for 2002 and 2003 was obtained from the Italian Institute of Statistics (ISTAT), which registers the national and regional population, by age group, as of the 1st of January for each year [14].

### Diagnoses/Complications

A listing was made of all main and secondary discharge diagnoses recorded in measles hospitalizations.

For the purposes of this study, non complicated measles cases were defined as cases with a main discharge diagnosis of "measles without mention of complication" (code 055.9) and without any secondary diagnoses. In the remaining cases, the type and frequency of complications were assessed by taking into account both main and secondary diagnoses. In addition to measles-specific codes, we included in the complications analysis other recorded ICD9-CM codes that represented possible complications of measles (see Additional File 1: ICD9-CM codes recorded as main and secondary discharge diagnoses in measles hospitalizations, Italy 2002–2003). In fact, ICD9-CM codes for complicated measles (055.0–055.8) do not include all possible complications of the disease; for example, thrombocytopenia is a possible complication of measles but there is no specific ICD9-CM code for this clinical condition when it occurs in a patient with measles.

### Costs

The costs of measles hospitalizations, at the national level and for each region, were estimated by use of DRG-based cost per case. The DRG system is a classification system designed to group hospital patients into categories which are clinically homogeneous with regard to the consumption of hospital resources. It is used, among other things, as a system of payment for the operating costs of hospital inpatient stays, based on predetermined rates [15]. Upon discharge from hospital, each patient is classified into a DRG on the basis of clinical information provided on the discharge abstract form. Each DRG has a payment weight assigned to it, based on the average resources used to treat patients in that DRG. Except for certain patients with exceptionally high costs (called outliers), the hospital is then paid a flat rate for the DRG. Separate rates exist for 1-day, ordinary (that is, with a length of stay > 1 day but < threshold value which defines outlier admissions) and outlier admissions. Because each of the 21 Italian regions establishes its own DRG rates, these of course vary between regions. For this reason, we used the rates agreed upon by all regions for the inter-regional reimbursement scheme regarding patients hospitalized out of their own region [16].

For the present study, we listed all DRGs attributed to measles-related admissions in 2002–2003, in each region, and calculated the number of 1-day, ordinary, and outlier admissions within each specific DRG. By using the above-mentioned DRG rates, we then calculated the costs of measles-related admissions.

### Data Analysis

Discharge abstract data were analyzed to obtain the following information:

- number and rates of measles hospitalizations per 100,000 population, by age group, for 2002 and 2003. Direct age-standardized hospitalization rates were calculated, by region and geographical area (North, Centre, South), using the Italian mean population of the two years combined as the standard population

- median age of cases

- distribution of diagnoses by age group

- length of hospital stay

- costs of measles hospitalizations, mean cost per admission day and mean cost per hospitalized case.

Categorical variables were compared using the χ^2 ^test. The Kruskal Wallis test was used to compare continuous variables. Chi square for trend test was performed to evaluate the linear association between diagnoses and age groups.

## Results

A total of 5,154 measles-related hospitalizations were identified, 3,072 of which occurred in 2002 and 2,082 in 2003. The median age of cases was 9 years (range 0–92 years).

### Main discharge diagnosis

Measles was registered as the main discharge diagnosis in most hospitalizations (Table 1). This proportion was similar in the three geographical areas (North 87.2%, Centre 89.6%, South 86.6%, p = 0.05).

**Table 1:** Main discharge diagnoses in measles hospitalizations. Italy, 2002–2003

**Main Diagnosis**	**N. hospitalizations (%)**
Measles (ICD9-CM codes 055.0–055.9)	4496 (87.2)
Respiratory infections or insufficiency	211 (4.1)
Other symptoms/conditions compatible with measles complications	246 (4.8)
Other medical conditions*	201 (3.9)
**Total**	**5154 (100.0)**

* including tumours, immune deficiency disorders, diabetes, other acute/chronic disorders

In approximately 70% of cases where measles was registered as a secondary diagnosis, the main diagnosis was respiratory infections/insufficiency or other symptoms/conditions compatible with measles complications (Table 1).

No statistically significant differences were observed in the median age of cases with and without a main discharge diagnosis of measles (respectively 9 and 7 yrs, p = 0.12). In both groups, approximately 52% of patients were male.

### Age-specific rates of hospitalizations

The hospitalization rate was 1.5 times higher in 2002 than in 2003 (Table 2). Most hospitalizations occurred in children <15 years of age (67.5%). In both years, children < 1 year of age had the highest hospitalization rates and rates decreased with increasing age.

**Table 2:** Age distribution of measles hospitalizations. Italy, 2002–2003.

**Age group (years)**	**2002**		**2003**	
	
	**N. hospitalizations (%)**	**N. hospitalizations/100,000 population**	**N. hospitalizations (%)**	**N. hospitalizations/100,000 population**
<1	241 (7.8)	46.2	102 (4.9)	19.0
1 – 4	834 (27.1)	39.8	382 (18.3)	17.9
5 – 9	781 (25.4)	29.2	379 (18.2)	14.3
10 – 14	430 (14.0)	15.3	329 (15.8)	11.6
15 – 19	258 (8.4)	8.8	313 (15.0)	10.8
≥ 20	528 (17.2)	1.1	577 (27.7)	1.2
**Total**	**3072 (100.0)**	**5.4**	**2082 (100.0)**	**3.6**

Note: All differences observed among age-groups were statistically significant (p < 0.05), with the exception of: 2003, age groups < 1 vs. 1–4 years and age groups 10–14 vs. 15–19 years.

### Hospitalization rates by region and geographical area

**Figure 1:**
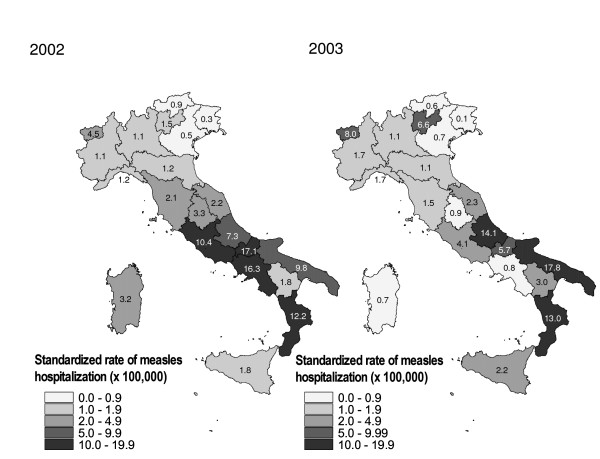
Standardised rate of measles hospitalizations per 100,000 inhabitants, by region. Italy 2002 and 2003.

Figure 1 shows the regional standardized hospitalization rates/100,000 population, for 2002 and 2003. In both years, over 71% of hospitalizations occurred in southern Italy, where hospitalization rates were found to be 5–9 times greater than in northern Italy (Table 3). Differences were especially marked in children below 10 years of age, with 5–21-fold higher incidence rates in southern with respect to northern regions. By contrast, the observed gap between northern and southern rates was smallest (3–4-fold) in the over-19 years age group.

**Table 3:** Age-specific measles hospitalization rates by geographical area. Italy 2002 and 2003.

	**N. hospitalizations/100 000 population**					
	
**Age group (yrs)**	**2002**			**2003**		
	*North*	*Centre*	*South*	*North*	*Centre*	*South*

**<1**	4.4	49.2	90.7	6.4	15.3	35.1
**1–4**	5.2	36.0	78.2	3.6	10.4	37.2
**5–9**	4.6	28.4	52.5	2.7	8.2	28.4
**10–14**	3.1	19.5	23.9	2.8	7.5	20.8
**15–19**	2.0	13.5	12.5	5.0	8.2	16.8
**≥ 20**	0.4	2.0	1.7	0.6	2.1	2.0
**Total**	**1.0**	**6.3**	**9.4**	**1.2**	**2.8**	**6.5**

Note: For each age group, differences observed among geographical areas were all statistically significant (p < 0.001).

### Diagnoses/Complications

Of 5,154 hospitalizations, 2,390 (46.4%) had a single discharge diagnosis of "measles without mention of complication" (ICD9-CM code 055.9). The remaining 2,764 hospitalizations included admissions with either a single discharge diagnosis of complicated measles (ICD9-CM codes 055.0–055.8: 471 admissions) or with two or more discharge diagnoses (2,293 admissions).

Table 4 shows the age distribution of clinical discharge diagnoses (N. = 6,496) reported for all hospitalizations. Over 50% of admissions involving individuals > 15 years of age were for non-complicated measles. Pneumonia was the most common complication and, together with otitis media and other respiratory tract conditions, was found to be more frequent in children < 10 years of age. On the other hand, encephalitis was more frequent in adolescents and adults.

**Table 4:** Diagnoses in measles hospitalizations, by age. Italy 2002–2003.

	Age group in years							
	
	**<1**	**1–4**	**5–9**	**10–14**	**15–19**	**≥ 20**	**Total**	***p*-value**
**N. Hospitalizations**	**343**	**1216**	**1160**	**759**	**571**	**1105**	**5154**	

**Non-complicated measles (%)**	150 (43.7)	506 (41.6)	486 (41.9)	375 (49.4)	315 (55.2)	558 (50.5)	2390 (46.4)	<0.001
**Pneumonia (%)**	48 (14.0)	198 (16.3)	176 (15.2)	60 (7.9)	34 (5.9)	78 (7.0)	594 (11.5)	<0.001
**Other respiratory tract complications (%)**	49 (14.3)	144 (11.8)	135 (11.6)	79 (10.4)	56 (9.8)	88 (8.0)	551 (10.7)	<0.001
**Enteritis/diarrhea/volume depletion (%)**	23 (6.7)	125 (10.3)	115 (9.9)	59 (7.7)	34 (5.9)	156 (14.1)	512 (9.9)	0.006
**Encephalitis (%)**	4 (1.2)	18 (1.5)	37 (3.2)	30 (3.9)	17 (3.0)	32 (2.9)	138 (2.7)	0.016
**Convulsions (%)**	7 (2.0)	70 (5.7)	16 (1.4)	7 (0.9)	1 (0.2)	0 (0.0)	101 (2.0)	<0.001
**Otitis media (%)**	10 (2.9)	36 (3.0)	25 (2.2)	12 (1.6)	6 (1.0)	12 (1.1)	101 (2.0)	<0.001
**Conjunctivitis/keratoconjunctivitis (%)**	4 (1.2)	15 (1.2)	21 (1.8)	14 (1.8)	16 (2.8)	38 (3.4)	108 (2.1)	<0.001
**Thrombocytopenia (%)**	2 (0.6)	3 (0.2)	3 (0.2)	7 (0.9)	5 (0.9)	6 (0.5)	26 (0.5)	0.170
**Abortion/threatened abortion/antepartum or fetal complication (%)**	1 (0.3)	0 (0.0)	0 (0.0)	0 (0.0)	2 (0.3)	17 (1.5)	20 (0.4)	<0.001
**Other infections/septicemia (%)**	16 (4.7)	48 (3.9)	47 (4.0)	25 (3.3)	17 (3.0)	57 (5.1)	210 (4.1)	0.514
**Other measles complications (%)**	54 (15.7)	142 (11.7)	161 (13.9)	112 (14.7)	117 (20.5)	259 (23.4)	846 (16.4)	<0.001
**Other diagnoses (%)**	50 (14.8)	170 (14.0)	170 (14.6)	121 (15.8)	75 (13.1)	314 (28.4)	899 (17.5)	<0.001
**Total N. Diagnoses**	**418**	**1475**	**1392**	**901**	**695**	**1615**	**6496**	

Note: For each diagnosis, percentages are calculated over the number of hospitalizations in each age group. Total percentages for each column exceed 100 because more than one discharge diagnosis may be listed for each hospitalization.

Of note are the 20 listed diagnoses of spontaneous or threatened abortion or other antepartum or fetal complications. The category "other measles complications" includes conditions (e.g. myocarditis, pericarditis) listed with a frequency of less than 20 diagnoses each.

Two deaths were registered in the Campania region, involving two female children aged respectively < 1 year and 11 years. Both deaths occurred as a result of respiratory insufficiency.

### Length of stay

Total admission time for the 5,154 measles hospitalizations was 27,017 days. The median length of stay was 4 days (range 1 – 303 days) and approximately 95% of admissions lasted ≤ 10 days. Median length of stay was 4 days in all age groups ≤ 19 years, and 5 days in cases aged >19 years (p < 0.001).

### Costs

Total hospital charges for the 5,154 admissions were approximately € 8.8 million (Table 5). The payer of hospital expenses was the National Healthcare Service (NHS) in 98.8% of cases (5,090 admissions).

**Table 5:** Length of stay and costs of measles hospitalizations, by geographical area. Italy 2002–2003.

**Geographical Area**	**N. hospitalizations**	**Median length of stay (days)**	**Total cost (€)**	**Mean cost per hospital day (€)**	**Mean cost per hospitalized case (€)**
North	541	4	1,066,723	338.97	1971.76
Centre	946	5	1,755,038	304.75	1855.22
South	3667	4	6,011,529	331.93	1639.36
**ITALY**	**5154**	**4**	**8,833,302**	**326.95**	**1700.14**

Each hospitalization cost, on average, an estimated €1,700. Non complicated measles cases cost on average €1,429 while the average cost per case of all other hospitalizations was €1,960. An even higher mean cost per case (€2,721) was observed in hospitalizations with a single discharge diagnosis of complicated measles (ICD9-CM codes 055.0–055.8).

Each admission day cost an estimated €327. The mean costs per hospital day and per hospitalized case were found to be consistent in the three different geographical areas (North, Centre, South).

## Discussion

Measles is now a rare disease in industrialised countries, and measles elimination targets have been set in the various World Health Organization (WHO) regions, such as Europe and the Americas; nevertheless, measles outbreaks continue to occur in under-vaccinated populations [17]. This study represents an important source of data regarding the health impact and economic costs of measles hospitalizations during a measles outbreak in Italy. Data was obtained from the national discharge abstract database, which has been shown to be >85% sensitive in assessing the frequency of disease conditions requiring hospitalization [18]. This type of database is available in a number of countries and, in countries using DRG costs to finance hospitals, has also been shown to be a reliable source of cost information for calculating the total cost of patient stays [19]. DRGs or similar grouping systems are well established in the USA and Australia and, over the past twenty years have been introduced in most European countries as instruments for reimbursement of hospital costs [20].

Recent studies have estimated the economic costs of measles cases and measles control in industrialized countries, mainly on the basis of various assumptions regarding measles incidence, vaccination coverage, hospitalization rates and treatment costs in different countries [11,21-24]. However, to our knowledge no nation- wide studies have been performed to directly evaluate the costs of measles-related hospitalizations on the basis of DRG rates.

Over two years, total direct hospitalization costs in Italy were estimated to be approximately €8.8 million, 98.8% of which (€8.7 million) were incurred by the NHS. The average cost of a hospitalized case, from the NHS perspective, is consistent with that estimated in another national study [24]. Costs per hospitalized case and per hospitalization day were comparable to those estimated for Canada, but higher than those estimated in two other European countries (Netherlands and the UK) [22]. This reflects the variability of hospital costs in different countries and may indicate the existence of higher hospital costs in Italy compared to other European countries.

It is well known that, in economic terms, the main advantage of measles vaccination is represented by the savings that may be achieved by preventing measles cases [23,25,26]. In Italy, vaccination coverage remains sub-optimal, and higher per capita measles costs have been reported, with respect to countries with better measles control [21]. Hospitalizations have been estimated to account for 40–50% of direct Italian NHS costs of measles cases [24], which also include primary care and medications. In 2002–2003, therefore, total direct measles costs in Italy may be considered to have been between €17.6 – 22.0 million. Approximately 97% of MMR vaccine is purchased and provided by the NHS [27] and the mean cost of one dose of vaccine in 2002 was €11.5. With the direct measles costs incurred by the Italian NHS in 2002–2003, therefore, approximately 1.5 to 1.9 million children, equivalent to 95% of 3 to 4 birth cohorts, could have been vaccinated with one dose of MMR vaccine, and many measles cases and hospitalizations may have been avoided. Administration of MMR would additionally have prevented many mumps and rubella cases and their related costs.

This outbreak caused more than 5,000 hospital admissions over two years. Close to 90% of these hospitalizations had measles as a main discharge diagnosis, and no significant differences in terms of age and geographical distribution were found between these cases and those with measles as a secondary diagnosis. Most of the latter cases had a main diagnosis of respiratory infections or other conditions compatible with measles complications, while only a minority of hospitalizations had a main diagnosis that was not related to measles.

Almost 50% of patients were hospitalized for non complicated measles. Although we cannot precisely identify the reason for admission in these cases, nor can we exclude that at least part of these admissions may have been inappropriate, these findings confirm that measles can be severe enough to require hospitalization even in industrialised countries and when specific complications are not present [7].

Over 65% of hospitalizations occurred in children aged 0–14 years and the greatest hospitalization rate occurred in children below 1 year of age. This was to be expected since, in the presence of suboptimal vaccine coverage, most measles cases continue to occur in small children and the disease is generally more severe in infants [28].

A large number of pneumonia and encephalitis cases were identified. The latter should be underscored, since long term sequelae of measles encephalitis are reported to occur in 20–30% of cases [29,30]; this implies that between 28 to 41 of the 138 encephalitis cases may have subsequently developed permanent disabilities. Unfortunately, we do not have data on the outcome of these cases. Finally, even though the number of deaths was small if compared to other causes of death, it must be underlined that these are preventable deaths that occurred in small children.

This study presents several limitations. The first limitation is that examined data refers to hospitalized cases with a discharge diagnosis of measles made on clinical grounds, without laboratory confirmation of infection. It is possible that some of these cases may have been misdiagnosed and that the total number of measles-related hospitalizations may therefore have been overestimated. Nevertheless, the positive predictive value of a clinical diagnosis of measles is higher in an epidemic period than in periods of low incidence [31].

A second limitation of the study is due to possible coding errors present in the discharge abstract forms. The hospital discharge abstract database relies on translation of physician discharge diagnoses into ICD9-CM codes, and different physicians may code for a specific diagnosis in different ways. Hospital medical charts were not reviewed in this study. It is possible, therefore, that at least some measles-related diagnoses may have been missed because codified with codes other than those relating to measles. For example, a total of 4 measles deaths were reported in 2002 in the Campania region [32] while only 2 appeared in the database. Although it is possible that the two additional deaths may have occurred outside the hospital setting, they also may have been coded with a discharge diagnosis other than measles.

A third limitation is represented by the fact that individual identification data for each admitted patient could not be sent to us for privacy reasons and so we were not able to identify patients who had been admitted more than once for the same condition. This, however, did not in any way affect our cost calculations nor did it affect calculations regarding the number of admission days and the median length of stay.

## Conclusion

The findings of this study highlight the substantial nationwide health burden associated with measles during the 2002–2003 outbreak, and its related costs. The study also shows how, in countries using DRG costs to finance hospitals, these may be used to calculate the cost of inpatient stays for a specific disease.

Measles hospitalizations in Italy in 2002–2003 mainly occurred in central and southern regions. This reflects the different regional vaccination strategies adopted in the past which have led, with a few exceptions, to higher coverage levels in northern regions with respect to the rest of the country [2]. A seroprevalence survey conducted in the late 1990s found a significantly higher proportion of children susceptible to measles in regions with coverage rates below 70%, and these regions were prevalently located in central and southern Italy [3,33].

In recent years, various actions aimed at improving coverage rates for measles and reducing regional differences have been undertaken at the national level. In 2002, MMR vaccination was included in the list of "essential health interventions" that all regions must provide free of charge within the NHS [34] and in 2003, the National Plan for the Elimination of Measles and Congenital Rubella was launched. Key strategies of the plan include programmes to achieve and sustain high coverage with 2 doses of MMR vaccine administered at 12–15 months and 5–6 years of age, implement a nationwide 'catch-up' vaccination campaign, and strengthen measles surveillance.

Since implementation of the elimination plan, MMR coverage rates in children by 2 years of age have been improving across Italy, and in 2005 reached 87%. In the same year a record low number of 108 measles cases were reported. Substantial progress has therefore been made towards the national elimination targets. However, in 2006, various clusters of measles cases occurred in under-vaccinated Roma/Sinti populations in different Italian regions [35]. These clusters are reminders that special attention is warranted for underserved population groups that have few or no contact with the health care system.

Additional efforts are still needed, therefore, to reach the WHO measles elimination target set for 2010 in the European region. Evidence based recommendations for increasing coverage of universal vaccinations, such as reminder and/or recall systems by healthcare providers and provider-based interventions for addressing missed opportunities should be reinforced. The high cost of measles and the severity of its complications fully justify the measles elimination effort.

## Competing interests

The authors declare that they have no competing interests.

## Authors' contributions

AF and MLCdA designed the study, reviewed the literature, collected, analyzed and interpreted the data, directed the project and were responsible for writing the manuscript.

AB reviewed the analysis plan, results and paper and provided economical expertise throughout the project.

AP reviewed the analysis plan, results, and manuscript.

MM and GMC performed the analysis.

All authors read and approved the final manuscript.

## Pre-publication history

The pre-publication history for this paper can be accessed here:



## Supplementary Material

Additional file 1 ICD9-CM codes measles hosp Italy. ICD9-CM codes recorded as main and secondary discharge diagnoses in measles hospitalizations, Italy 2002–2003. see title Click here for file
