# Supplementary material for: Health burden and economic impact of measles-related hospitalizations in Italy in 2002–2003
Source: BMC Public Health. 2007 Jul 24;7:169. doi: 10.1186/1471-2458-7-169 (PMC1963450; doi:10.1186/1471-2458-7-169)
Supplement: Additional file 1 — ICD9-CM codes measles hosp Italy. ICD9-CM codes recorded as main and secondary discharge diagnoses in measles hospitalizations, Italy 2002–2003. see title [file 1471-2458-7-169-S1.doc]

# Additional file 1

**ICD9-CM codes recorded as main and secondary discharge diagnoses in measles hospitalizations, Italy 2002-2003**

**Non complicated measles**: 055.9

**Pneumonia**: 055.1, 480, 480.8, 480.9, 481, 482.4, 482.89, 482.9, 483.0, 484.1, 484.8, 485, 486, 517.8

**Other respiratory tract complications**: 034.0, 460, 461.0, 461.1, 461.2, 461.8, 461.9, 462, 463, 464.0, 464.10, 464.11, 464.20, 464.21, 464.30, 464.4, 465.0, 465.8, 465.9, 466.0, 466.11, 466.19, 472.0, 473.0, 473.9, 474.11, 475, 478.0, 478.29, 478.75, 490, 491.21, 493.00, 493.01, 493.10, 493.11, 493.90, 493.91, 511.0, 511.8, 511.9, 512.0, 513.0, 514, 515, 516.8, 516.9, 518.81, 518.82, 518.89, 519.1, 770.8, 786.00, 786.09, 786.2, 786.3

**Enteritis/diarrhoea/volume depletion**: 003.0, 003.9, 005.9, 006.0, 007.1, 008.45, 008.5, 008.61, 008.62, 008.69, 008.8, 009.0, 009.1, 009.2, 009.3, 276.0, 276.1, 276.2, 276.5, 276.7, 276.8, 276.9, 536.2,558.2, 558.9, 787.01, 787.03, 787.91, 788.5

**Encephalitis**: 055.0, 323.0, 323.9

**Convulsions**: 780.3

**Otitis media**: 055.2, 381.00, 381.01, 381.02, 381.4, 382.00, 382.01, 382.4, 382.9 **Conjunctivitis/keratoconjunctivitis**: 055.71, 077.4, 077.99, 370.21, 370.40, 370.9, 372.00, 372.03, 372.30, 372.72

**Thrombocytopenia**: 287.4, 287.5

**Abortion/threatened abortion/antepartum or fetal complications:** 634.00, 634.90, 634.92, 641.90, 643.00, 643.83, 643.93, 644.00, 644.13, 646.60, 646.93, 647.60, 647.63, 648.93, 655.30, 765.07

**Other infections/septicaemia**: 041.00, 041.01, 041.04, 041.10, 041.19, 041.3, 041.4, 041.6, 041.7, 041.81, 041.84, 042, 047.9, 052.8, 052.9, 053.9, 054.10, 054.3, 054.9, 056.9, 057.8, 057.9, 070.1, 070.32, 070.54, 072.9, 074.0, 074.8, 075, 078.5, 078.88, 078.89, 079.0, 079.89, 079.98, 079.99, 081.9, 088.0, 110.5, 111.9, 112.0, 112.1, 112.2, 112.3, 112.4, 112.89, 112.9, 116.0, 127.4, 130.9, 132.0, 132.9, 133.0, 136.3, 136.9, 320.9, 322.2, 670.00, 684, 685.0, 686.0, 686.9, 771.0, 771.7, 771.8, 790.7, 998.59

**Other measles complications**: 055.79, 055.8, 049.8, 054.2, 070.59, 070.9, 074.3, 263.8, 307.81, 346.90, 348.3, 348.9, 359.9, 383.00, 383.9, 384.09, 388.70, 420.0, 420.90, 420.91, 422.0, 422.90, 422.91, 422.92, 423.9, 518.1, 528.0, 528.1, 528.2, 528.3, 540.0, 540.1, 540.9, 541, 542, 570, 573.1, 573.2, 573.3, 573.8, 573.9, 577.0, 580.0, 580.9, 583.2, 583.9, 584.5, 584.9, 590.10, 683, 711.50, 780.6, 780.7, 782.1, 783.0, 784.0, 785.6, 789.00, 789.06, 789.07, 789.09, 789.1

**Note:** All other ICD9-CM codes recorded were classified as “Other diagnoses”. These include tumours, other acute/chronic disorders, etc.
